# The Yellow Fever Panic

**Published:** 1888-12

**Authors:** J. C. LeHardy

**Affiliations:** Savannah, Ga.


### THE YELLOW FEVER PANIC.

BY J. C. LEHARDY, M. D., SAVANNAH, GA.

The panic occasioned by the announcement of yellow fever in Jacksonville, Fla., having sufficiently subsided to allow our people to come back to reason, I think the time opportune to discuss with them the causes which have produced this great disturbance of the public mind, and to show, if possible, how such panics may be prevented in the future.

The subject of this paper being of general interest, I have avoided the use of technical terms, and it may perhaps be well to premise, as an introduction to those not personally acquainted with me, that I am thoroughly and practically acquainted with yellow fever, having lived through the epidemics of 1854, 1858, and 1876, besides attending numbers of cases in the years intervening.

This practical knowledge, together with a careful reading of the history of yellow fever in the United States, has convinced me:

1st. That this is a disease which belongs essentially to the coast of the Atlantic and Gulf, and that for the last two centuries or more it has never been epidemic in any single year over any great extent of that coast. To illustrate, in 1876, when it raged so violently in Savannah, neither Charleston nor Jacksonville suffered in the least. In 1877, when Fernandina had her epidemic, the disease did not extend to the Georgia coast, while the epidemic of New Orleans in 1878, did not affect Florida or Georgia, *although little or no restrictions were put upon travel.*

2d. That an epidemic of yellow fever may extend throughout the valley of a river. In 1854 the epidemic of Augusta was co-existent with that of Savannah. In 1878 the epidemic of New Orleans extended far up the Mississippi Valley and its tributaries, and decimated many towns and villages along the course of these rivers.

3d. That it may be restricted to a portion of a valley. Such was the case in 1879, when the epidemic spent its force in Memphis and the surrounding country, while New Orleans remained perfectly healthy; and in 1882, when the epidemic of Brownsville was restricted to a portion of the Rio Grande.

4th. That it may be confined to a locality—a single village or a portion of a city, and there run its course in the same manner as when it involves the whole city. This occurred in Savannah, in 1858, in 1864, and in 1872; in and about Brewton, Fla., at Biloxi, Miss. (where the fever was called "*Spanish fever*"), at Tampa, Manatee, Plant City and other places.

It is evident from my experience and from accounts given by writers living in places subject to yellow fever, that whenever an epidemic occurs, the season is a *peculiar one.* Before the appearance of the fever the rainfall is *excessive*, and the solar heat very oppressive—always spoken of as something "*indescribable.*" Yellow fever is therefore a summer disease. It is also self-limited—an epidemic rarely begins before August, and rarely lasts, as such, after November. These are some of the features by which yellow fever has always been distinguished from other epidemic diseases in this country.

Asiatic cholera follows the line of travel, whether among the mountains or in the valleys, on the coast or in the interior; while small-pox will be communicated from individual to individual, wherever it may start, regardless of latitude, of climate, or of the season of the year.

The reason for these differences is that:

Yellow fever is produced by the *inhalation* of the germs (spores) of a plant which float in the air of an *infected place.*

Cholera is produced by the *drinking* of water in which the germs of the plant producing the disease are in *active existence.*

In small-pox the germs *emanate* from the individual suffering with the disease, so that whoever comes near him may be poisoned. For this reason small-pox is called contagious, and the others infectious.

In small-pox the germs emanate from the eruption. In cholera they emanate from the discharges of the bowels or the vomit, and are found in the water contaminated by these discharges. The germ which produces the yellow fever has not yet been demonstrated in the human body. So far we know the plant only by its *habits*, observed for the past 250 years. It will only propagate and fructify abundantly along the rivers and the coasts of the Atlantic and Gulf, because in these places only, will it find the *soil*, the *moisture* and *the heat* which it needs.

The reason why yellow fever never exists as an epidemic along any extensive coast line is that the conditions of earth and air required for the growth of the plant, never occur at the same time *along any extensive coast line.*

The reason that it will extend as an epidemic along the course of a river or in a valley, is that these conditions of earth and air *do sometimes exist* in every part of that valley at the same time.

From these premises, from my experience, and from the teachings of those who have acquired the greatest reputation for their knowledge of yellow fever, it would seem to me that the only conditions which should cause among our people a reasonable fear of an outbreak of yellow fever are:

1st. The situation of the city or village upon the Atlantic or Gulf coast, or in the valley of a river debouching thereon.

2d. The bad drainage of the place and its environs; a long continued hot spell and heavy rainfall in the early summer season. (Some sanitarians say, and perhaps correctly, "the presence of filth," though my own observations do not show that filth is necessary.)

3d. The coming of a ship from an infected port, of a closed car, a trunk or baggage, from an infected city—*the preceding conditions being present, but not otherwise.*

The seed (germ or spore) of the yellow fever plant may exist in the soil, or may hibernate from a preceding epidemic, in a closed building, a room, a vault or cellar, but it requires heat and moisture to bring it to active life and cause its fructification and spread. The imported germs require the same conditions, plus the soil, to enable them to grow and spread. *These conditions being wanting, they never can produce an epidemic.*

That certain conditions of the earth and air are absolutely necessary for the spread of yellow fever is proven by the fact that ships do come almost every year from infected places into our ports without producing an epidemic, or even a single case, provided the latter are in good sanitary condition.

It is well known that persons infected with the yellow fever germs have been imported in ships, or have taken refuge from infected localities, and have died from the "vomit," in Boston, New York, Philadelphia, Charleston, Savannah, New Orleans and many other places, without causing an epidemic. This could not possibly be the case with small-pox or any other contagious disease.

As stated before, yellow fever is not contagious from person to person, but must be contracted by the inhalation of air infected with the germs. Wherever the seed grows and fructifies, you have a focus of infection from which the surrounding air and soil become filled with spores (seeds). New plants are produced in every direction, the air becoming more and more *infectious*, so that a person coming within its influence, whether in-doors or out-doors, is liable to contract the disease. Thus it will spread so long as it meets the conditions of earth and air favorable to its growth, no matter how far that may be from the point of origin, *even over the most extensive valley;* but wherever these conditions cease, *there its growth is stopped*, the surrounding healthy air and soil prevent its spread like a wall of circumvallation, more effective than any "shot-gun quarantine"—called in civilized language "Cordon Sanitaire." That this is true, is proven by the partial epidemics at

Savannah in 1858, 1864, 1872, which affected only portions of the town, although no artificial means were used to confine them; and at New Orleans, Charleston and other places, at various dates. *But wherever the epidemic condition of earth and air is general,* all efforts to "*stamp out the disease have always proven utterly futile.*"

In a general epidemic there may be several points or foci of infection, widely separated from each other, in the same city or valley. When this is the case the circles of infection, starting from the foci, spread until they meet at various points, and so the whole city or valley becomes infected.

The concentration of the poison in the air goes on increasing until, at the height of the epidemic, it is so virulent that a person, whether acclimated or not, may contract the fever after a short time of exposure. This virulence is greatest inside of houses where the air is confined, so that a person visiting the sick is liable to take the disease; whence has arisen the general belief that yellow fever is contagious, when in fact it is only caused by exposure to a concentrated infection.

In 1876, at Savannah, a druggist, J. Polhill, who had had the fever early in the epidemic, went into a store in Yamacraw (one of the original points of infection) which had been closed when the epidemic was at its height, and within two hours was attacked with a violent case of yellow fever. He recovered. Toward the end of the epidemic (the place had been closed in the meantime), he repeated the experiment with a similar result. Shortly after the epidemic, two employees, returning from a protracted furlough, entered a room in the custom house, which had been closed during the epidemic, and both contracted yellow fever.

I have felt it necessary to be explicit and lengthy in laying down my propositions, because I believe that the panic now prevailing all over this country about yellow fever is altogether due to ignorance among the masses of the *only* conditions under which that disease can originate and spread. Prior to 1872, such a panic could not have been produced, the prevalent opinion at that time being that yellow fever *was not contagious,* and that an epidemic could originate in any place on the Atlantic coast, *with or without* importation of the germ. Consequently whenever the scourge did appear in a city, all those who could leave would do so, and they were received everywhere with open arms, no *fears* being entertained of its spread through "refugees."

The belief that the disease could be imported from the Barbadoes and other West India Islands induced our ancestors living on the coast to protect themselves against it by quarantine measures. From the early part of last century, quarantine was established at every port along the coast and at times rigidly enforced. These measures failed to prevent the recurrence of epidemics, and as they proved disastrous to commerce, the quarantine and sanitary conventions of 1857, '58, '59, '60 condemned them, and during the period of our civil war, and for some years afterwards, quarantine was nominal. Under this *régime*, strange as it may appear to some, no general epidemic occurred on the coast, although there were several outbreaks at *isolated points.*

Notwithstanding this immunity, a resolution was passed in Congress, in 1872, "providing for a more effective system of quarantine on the Southern and Gulf coasts," through the exertions of the advocates of the theory that yellow fever "is contagious" in its nature and of "exotic origin." Ever since that time quarantine has been held up before the people as a *sine qua non* in the prevention of yellow fever epidemics.

The supporters of this doctrine have impressed their theory in every possible way, through the public press, the medical journals, the American Public Health Association, etc., and have succeeded so well in turning the current of public opinion that they have induced our people to saddle themselves *de novo* with the old institution of quarantine, promising that if it was strictly enforced, no germ could enter and no epidemic ever occur in this country; sanitary measures were relegated by them to the back-ground as comparatively unimportant. The effect of this revolution is that the present generation hears little or nothing of the yellow fever *of the past;* it only knows it in its *new garb*, "*an exotic contagious disease.*

I may be able to illustrate the result of the teachings of this new doctrine by contrasting the condition of affairs at Savannah in 1876 and in 1888. The winter of 1875 and '76 was very mild ; delicate vegetation (coleus, geraniums, etc.) was not killed by the frost; stagnant water was covered with "green scum" as early as March. In June the heaviest rainfall ever recorded in Savannah occurred, viz., 18 inches within 10 wet days; the heat was *intense* and continued very oppressive through the whole summer. The city was filthy, the drainage neglected to such an extent that within and without the corporate limits pools and ponds of stagnant water, reaking with vegetation, were seen in every direction. The mortuary records reveal the fact that malarial fevers prevailed to an alarming extent during the spring and early summer—in fact, everything indicated the existence of *an epidemic condition of earth and air.* Yet the city authorities, although cognizant of the facts, remained perfectly inactive. Imbued with the theory so forcibly advanced at that time—that an epidemic of yellow fever could not possibly occur unless the germ was *imported*—they relied altogether upon *quarantine*, and they enforced it with vigor (*vide* report of the health officer for that year.) In June and July, however, cases of a suspicious fever appeared at various points in the city, and yellow fever was declared epidemic in the middle of August, when, as *usual*, the authorities attempted to *stamp out* the disease by disinfection, fires, etc., but *failed*, as they *always do.*

The failure of quarantine in preventing this epidemic—the untold losses caused by its ravages, forced the authorities, both of city and State, to institute systematic measures of sanitation, which have been kept up ever since, so that in 1888, after spending hundreds of thousands of dollars, they have succeeded in bringing Savannah and its environs into the cleanest and driest condition ever known in the history of the place. The season has been a remarkably favorable one; the rainfall by the middle of August was nearly *fifteen inches* below the average of the last *fifteen* years, and the solar heat was lacking 400 degrees of that average; malarial fevers have been *very scarce* and the mortality very small; yet with all this fair record before their eyes, the outbreak of yellow fever at Jacksonville has thrown the authorities into a state of unparalleled excitement because of the prevailing belief that the disease is *contagious.* Thousands of dollars have been spent in throwing lime about the streets and yards, in pouring disinfectants into every corner—other thousands in establishing and keeping up a system of inspection on all railroads and other avenues of approach. All travel and all commerce with Florida has been virtually stopped, and a system of passports, as onerous as in times of martial law, has been established, and this example, I am sorry to say, has been followed, not only by every town and village along the coast, but also by large and small communities all over the country. Shotgun quarantines, arrest, segregation in *pest houses* or islands, even forced labor on the chaingang and flogging have been visited upon innocent persons *because they were suspected of coming from infected places*. This is, to say the least, a sad reflection upon this enlightened age.

THE CAUSES OF THE PANIC.

Where must we look for the cause of this great retrogression of this senseless panic, and of these scenes and acts which would have been considered barbarous during the epidemics of the 14th century, when the dread plague destroyed hundreds of thousands of human beings—of those illegal procedures, which ought to bring shame into the breast of every thinking man in this great Republic—where ?

As stated before, ever since 1872 the advocates of the foreign origin and contagious nature of yellow fever have educated the public mind and imbued it with the correctness of their theory. Impelled by their urgings, the representatives of the people in Congress passed an act in 1877, '78 "to prevent the introduction of contagious and infectious diseases into the United States." A National Board of Health was next created, under whose direction quarantine stations were established along the coast; police boats were put on the Mississippi river, and the strictest set of rules ever enforced in this country, were put in operation. Inspectors were sent to various places—subsidized agents were placed in foreign ports to give warning of the existence of contagious diseases—to furnish trading vessels with bills of health, etc. The board spent all its energies upon the enforcement of a rigid quarantine on the coast and the Mississippi river, but failing to protect the people from the visitation of an epidemic of yellow fever in 1879, it and its supporters made the first attempt to establish inland (inter-state) quarantine, but the bill failed to pass through Congress.

The failure of the National Board to protect Memphis in 1879, *and its extravagant expenditure of money, destroyed the confidence* of the people in this "*great institution.*" Congress refused further appropriations for its support and relegated its powers to the Marine Hospital Service, which next undertook the task of preventing yellow fever epidemics by quarantine; but in 1882 an epidemic did occur in Brownsville and along the Rio Grande. During that epidemic, and ever since, it has been the policy of this service to expend its force in preventing the *spread* of epidemics.

Its method has been *isolation*, and its instruments camps for the sick, camps for the refugees, patrols, cordon *sanitaire* of inland quarantine, inspectors to examine persons, experts to examine suspicious cases and places, etc., all this under the sanction of State authorities.

The *apparent* success of its experiments on the Rio Grande in checking the spread of the epidemic in 1882, and afterward at Brewton and Biloxi, was trumpeted all over the country, and impressed the people with the belief that its measures were *certain preventives* and that any place *not adopting them was at the mercy of an epidemic*, through travel and commerce, no matter how great the distance from the point of infection.

To cap the climax, the straggling epidemic of Tampa in 1887, and the few suspicious cases which occurred late in the season at Plant City and Manatee, the telegrams and the reports of experts of the Marine Hospital Service, which were published in every newspaper in the land, hints of correspondents that yellow fever did exist at various places in Florida, and that the fact was kept secret by the municipal authorities of the State, all tended to keep the people on the "tender hooks" of apprehension, so that it only required the proclamation of yellow fever in Jacksonville to stir up the *fear*, which was soon to be intensified into a general panic by the efforts of the authorities of Savannah, and of every town and village along the coast, to establish an impassable quarantine; by the repeated telegrams and visits of the Surgeon-General of the Marine Hospital and Quarantine Service and of its experts, and by the many other manifestations of these authorities, calculated to make every man, woman and child feel that they were in great and imminent peril.

### THE PREVENTION.

In times of war and of pestilence, or during the prevalence of a panic, caused by the dread of some impending calamity, such as earthquakes, the defeat of armies, floods, cholera or yellow fever, which they see no means of averting, the people accept with blind faith anything which may promise the least chance of protection, and submit to any imposition which they are told by *those in authority* is necessary for their safety. It is self-evident that at such times there must be somewhere a power to direct, but it is also evident that such power is dangerous, because liable to be used arbitrarily by the ignorant or the designing.

In the present panic, caused by the dread of yellow fever *contagion*, quarantine is offered as the only means of affording protection, and the control of this *powerful engine* is put in the hands of a single man, who, like all mortals, is liable to be influenced by other motives than the public good.

The head of the quarantine service may never have seen a single case of yellow fever and know nothing of its nature, or he may be possessed with an inordinate desire to see himself lionized, or again, he may be a political aspirant, wishing to yield the power of a *national quarantine*, with its numerous refuge stations, its retinue of inspectors, of experts, etc., for his own private purposes.

Whatever may be the case in this respect at present, it is certain that a machine that can be used for evil as well as for good, should be guarded from falling into the hands of one man, or of many men, capable of being moved by selfish, by partisan, political or pecuniary considerations. The lesson of the present panic is, it would seem, that all matters pertaining to public health should be placed in the hands of a *scientific body*, which, like the

Weather Bureau or the Signal Service, is confined strictly to its own field of investigation and removed from all *political influences.* To this body should be intrusted the duty of collecting all possible information upon health and disease for the guidance of those in power. The scope of its investigations should not be restricted to contagious, infectious or any other class of diseases, but should include *everything* which can affect the health of the people. Its great object should be to increase the length of life by diminishing the mortality in a given population.

An examination of the following table, collated from the United States Census, will show that the mortality from our endemics—that is, diseases always among us, but too familiar to create dread—is so immensely greater than from yellow fever that it is difficult to understand why the latter has received so great a share of attention from the federal government, and why such large sums of money have been spent upon quarantine for its prevention, while no particular efforts have been made to devise means to reduce the mortality caused by the former class of diseases, which can be prevented by sanitary measures if properly carried out:

| CENSUS OF | 1850 | 1860 | 1870 | 1880 |
|---|---|---|---|---|
| POPULATION. | 23,191,876 | 31,443,321 | 38,555,983 | 50,155,783 |
| Mortality from all diseases | 358,626 | 443,208 | 498,170 | 756,893 |
| Consumption | 33,516 | 48,971 | 69,896 | 91,557 |
| Pneumonia | 12,130 | 27,076 | 40,012 | 65,565 |
| Scarlet Fever | 9,584 | 26,393 | 20,320 | 16,416 |
| Malarial Fever | 19,466 | 15,549 | 11,423 | 20,261 |
| Dysentery | 20,555 | 10,461 | 7,912 | 13,427 |
| Diphtheria | 00,000 | 1,663 | 6,303 | 38,308 |
| Yellow Fever | 787 | 657 | 177 | Not Rc'd |

The following will give the approximate mortality from:

| YELLOW FEVER | 1851 to 1860 | 1861 to 1870 | 1871 to 1880 |
|---|---|---|---|
| Total for 10 years | 26,843 | 10,132 | 12,738 |
| Annual ratio | 1,684 | 1,003 | ,274 |
| Ratio per 1,000 of Population | 0,015 | 0,031 | 0,035 |
| Ratio per 1,000 of all diseases | 7.49 | 2.28 | 2.55 |
| Ratio per 1,000 of Endemics | 16.01 | 4.34 | 5.07 |

Such a body as herein proposed would have for its sole aim the attainment of truth, based upon scientific and practical investigations. Its calm and dispassionate proceedings in considering all facts would commend it to the confidence of all those in authority and lead them to be guided by its decisions. Truth sustained by authority cannot fail to enlighten, and the popular mind would gradually be disabused of ignorance and strengthened against fear and panics. The present wasteful expenditure of energy and money would then be directed into efforts that would prove not only local preventives, but of permanent benefit to the *health of the nation.*

*October 13, 1888.*

---

EDITORIAL CHANGE.—Having accomplished all that he had hoped or expected to accomplish when he accepted the editorship of *The Journal of the American Medical Association* in 1883, and feeling the necessity for diminishing his responsibilities and constant work, the editor of *The Journal* tendered his resignation to the Board of Trustees in the latter part of June last, the same to take effect on the 31st day of December, 1888. At a special meeting of the Board of Trustees held in Chicago, November 9 and 10, 1888, the resignation was accepted, and John B. Hamilton, M. D., Supervising Surgeon-General of the Marine Hospital Service and late Secretary-General of the Ninth International Medical Congress, was chosen unanimously to fill the place. He will enter upon the discharge of his editorial duties January 1, 1889. We trust he will have a long and prosperous editorial career.—*N. S. Davis, M. D., in Journal American Medical Association.*

